# Association between serum zinc levels and basic physical functioning: secondary data analysis of NHANES 2011–14

**DOI:** 10.1186/s40795-021-00461-z

**Published:** 2021-10-11

**Authors:** Jen-Tzer Gau, Bhakti Chavan, Yang Li, Brian C. Clark, Zelalem T. Haile

**Affiliations:** 1 Department of Primary Care, Heritage College of Osteopathic Medicine (HCOM), Ohio University, Athens, OH 45701 USA; 2 Office of Research and Grants, HCOM, Athens, OH 45701 USA; 3 Department of Biomedical Sciences, HCOM, Athens, OH 45701 USA; 4 Department of Biomedical Sciences and Ohio Musculoskeletal and Neurological Institute, HCOM, Athens, OH 45701 USA; 5 Department of Social Medicine, HCOM, Dublin campus, Ohio University, Dublin, OH 43016 USA

**Keywords:** Zinc, Activities of daily living (ADL), Demography, Health surveys, Nutrition surveys

## Abstract

**Background:**

Serum zinc (Zn) levels have been shown to be associated with functional status; however, it is not clear whether this association differs by other sociodemographic characteristics. We examined the association between serum Zn levels and physical functioning difficulty in a representative sample of older adults in the US.

**Design and methods:**

A cross-sectional study was conducted on participants 50 years and older from the 2011–12 and 2013–14 National Health and Nutrition Examination Surveys (*n* = 1136). Serum Zn levels were analyzed as tertiles. The main outcome of interest was physical functioning difficulty, defined as self-reported difficulty of basic physical functioning that included walking, transferring, dressing, and feeding.

**Results:**

Mean Zn levels (SE) were 0.67(0.1), 0.81(0.1), and 0.98(0.1) μg/mL in the low, middle, and high Zn groups, respectively. Approximately 24.9% participants reported physical functioning difficulty. In the multivariable model, we found a significant multiplicative interaction between sex and serum Zn (P for interaction =0.028) and between education and serum Zn (P for interaction = 0.001) on basic physical functioning difficulty. The stratified analysis revealed that among men, compared to those with low serum Zn, the odds of having physical functioning difficulty were lower in men who had high serum Zn [aOR 0.43 (95% CI: 0.25–0.76)]. For women, compared to those with low serum Zn the odds of having physical functioning difficulty were higher in women who had middle serum Zn [aOR 2.67 (1.58–4.50)]. Among individuals with less than high school education, the odds of having physical functioning difficulty were lower in those who had middle serum Zn compared to those who had low serum Zn [aOR 0.48 (0.26–0.89)]. However, the odds of having physical functioning difficulty were higher in those who had middle serum Zn compared to those who had low serum Zn for individuals with high school [aOR 5.72 (1.92–17.00)] and beyond high school education [aOR 1.77 (1.05–2.97)], respectively.

**Conclusion:**

Sex and educational attainment interact with serum Zn levels to influence basic physical functioning difficulty in older adults.

**Supplementary Information:**

The online version contains supplementary material available at 10.1186/s40795-021-00461-z.

## Introduction

One in four non-institutionalized US adults (61 million, or 25.7%) report some form of physical disability [1]. People with disability have reduced life space that impacts their overall quality of life. Further, disability is a risk factor predicting mortality in older adults with adverse health events such as falls with fractures, nursing home admission, and death [2]. People with disability often require home health care services; the cost of these services reached US $108.9 billion in 2019 and is expected to increase to $201 billion by 2028 [3]. Identifying factors that influence or avert physical disability could enable strategies to reduce these negative impacts.

Optimal nutrition is essential for good health and facilitates recovery from acute illness. One factor contributing to these benefits is micronutrient concentration status [4–6]. For example, older women with low micronutrient levels are at increased risk for disability later in life [7]. Among many micronutrients, zinc (Zn) is an essential element commonly used by biological systems as a cofactor for enzymes and as a structural cofactor for many proteins [8]. Thus, unsurprisingly, serum Zn levels are linked with health outcomes including physical performance [8], the ability to concentrate [9–11] and aspects of musculoskeletal health [e.g., lower serum Zn levels are associated with osteoporosis [12], low bone mineral density [13], and are implicated in muscle weakness [14, 15].

Lower serum Zn levels are associated with a less diversified diet [16, 17], which increases the risk of malnutrition. People with malnutrition have increased odds of decline in activities of daily living (ADL) over time compared to those without malnutrition [18]. This likely explains why serum Zn levels have been associated with physical performance [19] and functional status [20, 21]. There are, however, conflicting findings [9, 22]. Additionally, it is unclear whether the association between serum Zn status and the physical functioning status varies by other sociodemographic characteristics. Serum Zn levels have been shown to be associated with sex [23, 24]. It is possible that the contribution of serum Zn on functional status may vary by sex.

The current study examined the association between serum Zn levels and basic physical functioning difficulty among adults aged 50 and older by combining National Health and Nutrition Examination Survey (NHANES) data from 2011 to 2014. We hypothesized that the association between serum Zn level and physical functioning difficulty would differ by sex and other sociodemographic characteristics (such as education).

## Materials and methods

### Study design and population

This cross-sectional study is based on secondary data from the 2011–12 and 2013–14 NHANES. NHANES, conducted by the National Center for Health Statistics, interviewed approximately 5000 individuals of all ages in their homes. The details of the design, target population, and content of the public use data files are described elsewhere [25]. Participants were selected using a stratified, multistage probability design. Hispanics, non-Hispanic Blacks, non-Hispanic/non-Black Asians, low-income non-Hispanic Whites, and persons 80 years or older were oversampled to provide stable estimates of these groups.

The present study sample consisted of 1247 NHANES 2011–12 and 2013–14 participants aged 50 years and older who had serum Zn level measurements and self-reported physical functioning information. We excluded participants with missing data (*n* = 111) on covariates included in the multivariable model. The final sample comprised 1136 participants. This study was approved by the Institutional Review Board.

### Basic physical functioning difficulty

In this study, basic physical functioning status is defined based on the four questions under the module “Physical Functioning” that are available in both NHANES 2011–12 and 2013–14 dataset in the elements of the Katz index of independence of basic ADL [26]: “Without using any special equipment, how much difficulty do you have walking from one room to another on the same level?”, “Without using any special equipment, how much difficulty do you have getting in or out of bed?”, “Without using any special equipment, how much difficulty do you have eating, such as holding a fork, cutting food or drinking from a glass?”, “Without using any special equipment, how much difficulty do you have dressing yourself, including tying shoes, working zippers, and doing buttons?” The response options to the question range from 1 to 5 to reflect no difficulty, some difficulty, much difficulty, unable to do, or do not do this activity, respectively. We coded the response as 0 if the participant reported performing an activity with no difficulty and responses were coded as 1 if the participant reported some difficulty, much difficulty, or “unable to do”. Following existing literature, participants who reported “do not do this activity” were excluded from the analysis [27]. The distribution of self-reported difficulty in four basic physical functioning was shown in supplementary Additional file 1: Table 1A and 1B. Because of the problems that were encountered with small sample sizes in some cells, an issue of convergence, and unstable odds ratio estimates, our study adopted the coding for the basic physical functioning status as having no difficulty vs. having difficulty (0 vs. 1). While there are well-known limitations of self-reported measures of physical functioning, they are reliable [28], and both men and women generally report their disability accurately [29, 30].

### Serum Zn concentrations

Serum Zn levels were measured by inductively coupled plasma dynamic reaction cell mass spectrometry (ICP-DRC-MS) method as described by NHANES 2011–14 protocols [31]. Participants were categorized into three groups by serum Zn tertile: low (range: 0.409–0.748 μg/mL, *n* = 404), middle (range: 0.749–0.870 μg/mL, *n* = 380), and high (range: 0.872–1.698 μg/mL, *n* = 352) group. Serum Zn levels in SI unit (μmol/L) can be converted by multiplying the factor 15.3. Serum albumin and blood hemoglobin concentrations which are significantly associated with serum Zn levels [23] were obtained from laboratory files of NHANES 2011–14 data [32].

### Covariates

Age, sex, race/ethnicity, and health insurance information was assessed using the questionnaire. Body mass index (BMI) calculated as weight in kilograms divided by height in meters squared was categorized into four groups: obese (BMI ≥ 30); overweight (BMI 25.0–29.9); normal (BMI 18.5–24.9); and underweight (BMI < 18.5). Race/ethnicity was based on self-reported information and was categorized into three groups: non-Hispanic white, non-Hispanic black, and others. Education was categorized into three groups: less than high school, high school, and more than high school.

Self-reported physical activity was categorized into two groups, non-sedentary vs. sedentary, based on the ‘yes’ vs. ‘no’ response to the question “Do you do any moderate-intensity sports, fitness, or recreational activities that cause a small increase in breathing or heart rate such as brisk walking, bicycling, swimming, or golf for at least 10 minutes continuously?” Participants were categorized as drinkers or non-drinkers based on self-reported alcohol use, defined as having at least one drink per week: “In the past 12 months, on those days that you drank alcoholic beverages, on average, how many drinks did you have?” Smoking status was categorized as never, former, and current. Current smokers were those who responded yes to the question “Do you now smoke cigarettes?” Former smoker and never smokers were categorized based on the response to questions “Have you smoked at least 100 cigarettes in your entire life?” and “Do you now smoke cigarettes?” Diagnosis of diabetes mellitus was based on self-reported response to the question “Have you ever been told by a doctor or health professional that you have diabetes or sugar diabetes?”

### Statistical analysis

Descriptive statistics were performed to describe and summarize the data. Using Rao-Scott chi-square and independent sample t-tests, bivariate analyses were performed to compare groups. We then used a multivariable logistic regression model to calculate the adjusted odds ratio (aOR) and 95% confidence interval (95% CI) for basic physical functioning difficulty by serum Zn level tertile, using the lowest tertile as the reference group. Pairwise interaction terms between serum Zn levels and covariates were included in the multivariable regression to examine subgroup differences in the association between serum Zn status and basic physical functioning difficulty. The covariates that were included in this study for the multivariable model regression were age, sex, BMI, race, education, status of health insurance, smoking, alcohol use, physical activity, diabetes, serum albumin, and blood hemoglobin levels. Covariates were retained in a multivariable model regardless of the results of univariable analyses. Potential multicollinearity was assessed using a variance inflation factor. Regression analysis was not prone to multicollinearity. Statistical significance was set at *p* ≤ 0.05. SAS® version 9.4 software (SAS Institute, Inc., Cary, NC) was used to perform all statistical analyses. All analyses incorporated sample weights that account for the unequal probabilities of selection, oversampling, nonresponse, and complex survey design elements of NHANES.

## Results

### Characteristics of NHANES 2011–14 participants by serum Zn status tertile

Table 1 summarizes study participant characteristics. Mean Zn levels (standard error, SE) were 0.67 (0.01), 0.81 (0.01), and 0.98 (0.01) μg/mL in the low, middle, and high Zn tertile groups, respectively. Lesser proportion of participants in high Zn tertile group reported basic physical functioning difficulty compared to the middle and low Zn tertile group, respectively (25.8% vs. 40% vs. 34.2%). Bivariate analyses found a significant association between serum Zn concentration level and basic physical functioning difficulty (*p* = 0.036), sex (*p* = 0.016), and BMI (*p* = 0.031). Participants with high Zn levels were more likely to be men, without physical functioning difficulty, and overweight. There were significant differences in the mean concentration of serum albumin and blood hemoglobin levels between the three Zn groups (*p* < 0.01). There were no significant differences in Zn levels and physical functioning status between those who were included in the study and those who were excluded due to missing covariate data (data not shown).

**Table 1:** Characteristics of participants aged 50 years and older in NHANES 2011–14 based on serum zinc concentration tertile (total *n* = 1136)

Variables^*+*^	Low Zn tertile (***N*** = 404)	Middle Zn tertile (N = 380)	High Zn tertile (N = 352)	P value
Mean Zn level (+/−SE) (μg/mL)§	0.67 (±0.01)	0.81 (±0.01)	0.98 (±0.01)	
range	0.409–0.748	0.749–0.870	0.872–1.698	
Basic physical functioning difficulty				0.036
Yes (*n* = 283)	102 (34.2%)	105 (40.0%)	76 (25.8%)	
No (*n* = 853)	302 (33.8%)	275 (30.7%)	276 (35.5%)	
Age (+/− SE) years	67.3+/− 0.5	66.9+/− 0.7	66.1+/− 0.4	0.186
(95% CI)	(66.2–68.4)	(65.5–68.4)	(65.4–66.9)	
Sex				0.016
Male (*n* = 561)	203 (32.5%)	172 (27.2%)	186 (40.4%)	
Female (*n* = 575)	201 (35.2%)	208 (37.7%)	166 (27.1%)	
BMI				
Under (*n* = 19)	6 (15.8%)^**+**^	10 (69.1%)^**+**^	3 (15.1%)^**+**^	0.031
Normal (*n* = 301)	108 (35.1%)	98 (33.3%)	95 (31.6%)	
Overweight (*n* = 400)	131 (29.5%)	136(33.0%)	133 (37.4%)	
Obese (*n* = 416)	159 (38.6%)	136(30.2%)	121 (31.2%)	
Race/ethnicity				0.516
Non-Hispanic White (*n* = 533)	186 (33.7%)	174(32.0%)	173 (34.3%)	
Non-Hispanic Black (*n* = 261)	102 (39.3%)	87 (31.4%)	72 (29.3%)	
Others (*n* = 342)	116 (31.8%)	119 (37.6%)	107 (30.6%)	
Education				0.786
< High school (n = 352)	125 (31.6%)	118(37.0%)	109 (31.5%)	
= High school (*n* = 253)	87 (34.0%)	83 (31.8%)	83 (34.2%)	
> High school (*n* = 531)	192 (34.6%)	179 (31.7%)	160 (33.7%)	
Heath insurance				0.599
Yes (*n* = 1001)	361 (34.5%)	339 (32.6%)	301 (32.9%)	
No (*n* = 135)	43(28.8%)	41 (33.4%)	51 (37.8%)	
Drinker				0.225
Yes (*n* = 605)	202 (33.6%)	219 (34.7%)	184 (31.7%)	
No (n = 531)	202 (34.4%)	161 (29.5%)	168 (36.1%)	
Smoking				0.483
Never (*n* = 541)	208 (36.5%)	179 (32.0%)	154 (31.5%)	
Former (*n* = 410)	128 (30.6%)	143 (33.9%)	139 (35.6%)	
Current (*n* = 185)	68 (34.2%)	58 (31.9%)	59 (33.9%)	
Physical activity				0.528
Yes (*n* = 405)	134 (31.8%)	135 (32.4%)	136 (35.8%)	
No (*n* = 731)	270 (35.3%)	245 (32.9%)	216 (31.8%)	
Diabetes				0.252
Yes (*n* = 277)	93 (30.7%)	89 (29.8%)	95 (39.5%)	
No (*n* = 859)	311 (34.8%)	291 (33.5%)	257(31.7%)	
Serum albumin^+^				< 0.01 between groups
(mean +/− SE) (gm/dL)	4.11+/−0.02	4.23+/−0.02	4.32+/−0.02	
(95% CI)	(4.07–4.14)	(4.19–4.27)	(4.27–4.37)	
Hemoglobin				< 0.001
(mean +/− SE) (gm/dL)	13.6+/−0.1	14.0+/− 0.1	14.1+/− 0.1	
(95% CI)	(13.4–13.8)	(13.8–14.1)	(14.1–14.6)	

^**+**^ All percentages reported here are based on a weighted percentage

§ converting to SI unit (μmol/L) by multiplying 15.3

^+^ converting to SI unit (g/L) by multiplying 10

Abbreviation: BMI = body mass index

### Characteristics of study participants with basic physical functioning difficulty

Approximately 24.9% of participants self-reported some form of basic physical functioning difficulty (Table 2); participants with 1 or 2 counts of difficulty (total *n* = 212) accounted for 75% (212/283) of those with physical functioning difficulty in our study sample (Additional file 1: Table 1B). A greater proportion of participants with difficulty were non-Hispanic black (28.1%) and others (26.6%) compared to non-Hispanic white (20.3%; *p* = 0.014). There was a significant difference in education level, smoking status, physical activity, self-reported diabetes, and mean hemoglobin levels between those with and without difficulty (all *p* values < 0.05). In the multivariable model, we found a significant multiplicative interaction between sex and serum Zn (p for interaction =0.028) and between education and serum Zn (p for interaction = 0.001) on basic physical functioning difficulty.

**Table 2:** Characteristics of NHANES 2011–14 participants aged 50 years and older with and without self-reported basic physical functioning difficulty

	Basic physical functioning difficulty		
Variables^*+*^	YES (N = 283; 24.9%)	NO (N = 853; 75.1%)	P value
Age (+/− SE) years	65.7 +/− 0.8	67.1 +/− 0.4	0.134
(95% CI)	(64.1–67.3)	(66.3–67.8)	
Sex			0.792
Male (n = 561)	133 (21.4%)	428 (78.6%)	
Female (n = 575)	150 (22.3%)	425 (77.7%)	
BMI			0.075
Under (n = 19)	9 (27.1%)^+^	10 (72.9%)^+^	
Normal (n = 301)	59 (18.0%)	242(82.0%)	
Overweight (n = 400)	89 (19.4%)	311 (80.6%)	
Obese (n = 416)	126 (27.1%)	290 (72.9%)	
Race/ethnicity			0.014
Non-Hispanic White (n = 533)	130 (20.3%)	403 (79.7%)	
Non-Hispanic Black (n = 261)	70 (28.1%)	191 (71.9%)	
Others (n = 342)	83 (26.6%)	259 (73.4%)	
Education			0.029
< High school (n = 352)	105 (27.3%)	247 (72.7%)	
= High school (n = 253)	66 (26.9%)	187 (73.1%)	
> High school (n = 531)	112 (18.3%)	419 (81.7%)	
Health insurance			0.852
Yes (n = 1001)	253 (21.8%)	748 (78.2%)	
No (n = 135)	30 (22.8%)	105 (77.2%)	
Drinker			0.068
Yes (n = 605)	129 (18.9%)	476 (81.1%)	
No (n = 531)	154 (26.6%)	377 (73.4%)	
Smoking			0.008
Never (n = 541)	120 (18.6%)	421 (81.4%)	
Former (n = 410)	108 (22.1%)	302 (77.9%)	
Current (n = 185)	55 (32.7%)	130 (67.3%)	
Physical activity			0.012
Non-sedentary (n = 405)	68 (15.5%)	337 (84.5%)	
Sedentary (n = 731)	215 (26.2%)	516 (73.8%)	
Diabetes			0.004
Yes (n = 277)	86 (31.7%)	191 (68.3%)	
No (n = 859)	197 (19.2%)	662 (80.8%)	
Serum albumin (gm/dL)§			0.088
(mean +/− SE)	4.16+/− 0.03	4.23+/−0.02	
Hemoglobin (gm/dL)			0.038
(mean +/− SE)	13.75 +/− 0.14	14.06 +/− 0.06	
(95% CI)	(13.46–14.04)	(13.93–14.18)	

^**+**^ All percentages reported here are based on a weighted percentage

§ converting to SI unit (g/L) by multiplying 10

Abbreviation: BMI = body mass index

### Association between serum Zn tertile status and basic physical functioning difficulty stratified by sex

Table 3 presents the association between serum Zn levels and physical functioning difficulty, stratified by sex. Among men, compared to those with low serum Zn, the odds of having physical functioning difficulty were lower in men who had high serum Zn [aOR 0.43 (95% CI: 0.25–0.76)]. For women, compared to those with low serum Zn the odds of having physical functioning difficulty were higher in women who had middle serum Zn [aOR 2.67 (1.58–4.50)]. Men and women had a different profile of risk factors for basic physical functioning difficulty (shown in Additional file 1: Table 2).

**Table 3:** Association between serum Zn status and basic physical functioning difficulty stratified by sex, 2011–14 NHANES survey data

Multivariable-adjusted† odds ratio with 95% confidence interval		
	Male (physical difficulty: yes = 133, No = 428); aOR (95% CI)	Female (physical difficulty: yes = 150, No = 425); aOR (95% CI)
**Serum Zn tertile**		
**High vs. low Zn group**	0.43 (0.25–0.76)*	**1.42 (0.76–2.65)**
**Middle vs. low Zn group**	**0.73 (0.35–1.52)**	2.67 (1.58–4.50)*

Note: Model was adjusted for age, weight (obesity vs. normal, overweight vs. normal, underweight vs. normal), race/ethnicity, education, health insurance, drinking status, smoking status, physical activity, diabetes, serum albumin (gm/dL), and blood hemoglobin (gm/dL)

* Indicates a significant association with p values < 0.05

† Variables except age, hemoglobin, and serum albumin level were dichotomized as 1 = yes, 0 = no (baseline)

Abbreviations: aOR = adjusted odds ratio; CI = confidence interval

### Association between serum Zn groups and basic physical functioning difficulty stratified by education attainment

Table 4 presents the association between serum Zn levels and physical functioning difficulty, within the subgroups of educational attainment. Among individuals with less than high school education, the odds of having physical functioning difficulty were lower in those who had middle serum Zn compared to those who had low serum Zn [aOR 0.48 (0.26–0.89)]. However, the odds of having physical functioning difficulty were higher in those who had middle serum Zn compared to those who had low serum Zn for individuals with high school [aOR 5.72 (1.92–17.00)] and beyond high school education [aOR 1.77 (1.05–2.97)], respectively.

**Table 4:** Association between serum Zn status and basic physical functioning difficulty stratified by education, 2011–14 NHANES survey data

Multivariable-adjusted odds ratio with 95% confidence interval			
	Stratified analyses		
	**Education**		
**Zn level status**	**< High school (N = 352)** **Physical functioning difficulty** Yes = 105; No = 247	**High school (N = 253)** **Physical functioning difficulty** Yes = 66; No = 187	**> High school (N = 531)** **Physical functioning difficulty** Yes = 112; No = 419
Low Zn tertile group	[Reference]	[Reference]	[Reference]
Middle Zn tertile group	0.48 (0.26–0.89)*	5.72 (1.92–17.00)*	1.77 (1.05–2.97)*
High Zn tertile group	0.51 (0.24–1.08)	2.42 (0.77–7.56)	0.65 (0.39–1.14)

Note: Model was adjusted for age, sex, weight (obesity vs. normal, overweight vs. normal, underweight vs. normal), race/ethnicity, health insurance, drinking status, smoking status, physical activity, diabetes, serum albumin (gm/dL), and blood hemoglobin (gm/dL)

Abbreviations: aOR = adjusted odds ratio; CI = confidence interval

* Indicates a significant association with *p* values < 0.05

## Discussion

We found an overall significant association between serum Zn levels and basic physical functioning difficulty in a nationally representative sample of US adults aged 50 years and older. The association varied based on sex and educational attainment and was independent of age, BMI, race/ethnicity, health insurance, alcohol use, smoking status, physical activity, serum albumin, and hemoglobin levels. In men, higher Zn levels were associated with lower odds of having physical functioning difficulty whereas the opposite trend was observed in women. Notably, participants with less than high school education showed a similar trend to that observed in men, of a negative association between higher Zn level and physical functioning difficulty. Our study findings further extend the previously reported associations between serum Zn and physical functional status and demonstrate that men and women have a different association trend between serum Zn and basic physical functioning difficulty.

The correlation between serum Zn concentration and physical functional status has been investigated by a few studies [9, 19–22]. A study of 103 New Zealand older women (mean age 75 ± 3 years) showed that those women who had higher tertile physical functioning score had significantly higher serum Zn concentrations, 12.9 μmol/L vs. 12.0 μmol/L in the lower tertile physical functioning score group, after adjusting for serum albumin and α-1-glycoprotein [19]. This study’s physical functioning score was based on the sum of the measurements of handgrip, quadriceps muscle strength, ADL, and “Timed up and go” results. A small study of long-term care facility residents revealed that Zn levels were correlated with low BMI, higher level of care needed, and higher grade of bed-riddenness [21]. However, a cross-sectional study of 82 older adults (average age 81 ± 8 years, 78% women) recruited from mental health centers and nursing homes reported no association between serum Zn concentration and ADL based on the Barthel Index score [9]. In our study, the basic physical functioning status is based on self-reported information, and approximately 75% of participants who self-reported physical functioning difficulty had reported only one or two counts of difficulty in the four aspects, i.e., walking/mobility, out of bed/transferring, feeding, and dressing (Additional file 1: Table 1A and 1B). With the adjustment of covariates including serum albumin and hemoglobin levels, our study revealed that sex and educational attainment interact with serum Zn levels to influence basic physical functioning difficulty in adults aged 50 and older.

Our study revealed inconsistent findings compared to the study of de Jong et al. [19]. There were differences between these two studies in study subjects’ profiles as well as in the measurement of physical functioning status. Our study analyzed those aged ≥50 years of a general population with multiple ethnic groups. The mean serum Zn concentration in each tertile group was quite a difference with a wider range, i.e., 0.67 μg/mL (≈10.25 μmol/L), 0.81 μg/mL (≈12.39 μmol/L), and 0.98 μg/mL (≈14.99 μmol/L) in each tertile Zn group, respectively. In contrast, the study of de Jong et al. mainly focused on older women aged 70 to 80 years of white people; many of the study participants (74%) reported fear of falling and only 14% of them able to manage all ADL. The mean Zn concentration had a narrower range between the tertile group of physical functioning scores, i.e., from 12.0 to 12.9 μmol/L [19]. Because the study subjects were different in health status and profiles, it would not be possible to make further comments on the result interpretation between these two studies.

The effect of sex on Zn homeostasis is complex. Zn is needed for normal development of the male reproductive system and sperm production and may play a role in modulating serum testosterone levels in men [24]. Some studies described differential effects of Zn between men and women, such as in serum leptin levels with Zn supplementation [33], association with metabolic syndrome [34], and prevalence of kidney stones [35]. Serum Zn concentrations are related to sex but not to dietary or supplemental Zn intake in the US population [23], which suggests a hormonal effect underlying Zn homeostasis.

It is plausible that people with different education levels, racial/ethnic groups, or professions have different lifestyle or dietary preferences that may have impacted on the serum Zn status as well as on self-reported physical functioning status. Because this study’s physical functioning status is based on self-reported information, our study could not exclude the possibility of sex or educational status playing a role in self-reporting physical or functional health status. Studies have shown that men than women under-reported disability status [29], or women have a tendency of reporting ADL or mobility disability status than their physical performance status based on examination [36] or observation [37]. Similarly, people with different educational status may report differently on their functioning status compared to their physical status. In the consideration of all these factors, searching for a biomarker that could represent the underlying confounders for outcome research will be needed. The complexity of our findings may suggest an alternative approach in analyzing the data in future studies that may include, but is not limited to, using the ratio of serum copper to Zn levels, which has been proposed as a potential biomarker for physical functional status [14, 15, 38, 39].

Zn intake from supplements has been increasing among middle-aged and older adults [23]. In fact, approximately 10 and 4% of men and women, respectively, aged > 71 years who were supplement users had a daily Zn intake exceeding 40 mg (the tolerable upper limit) [40]. Too much Zn supplementation decreases absorption of copper [41] and iron [42] and may affect magnesium balance [43].

Our study has some limitations. First, the association identified by our study could result from confounders that were not included in the multivariate logistic regression data analysis. Second, self-reported basic physical functioning may be over- or under-reporting true functioning status. Third, because responses were self-reported, the possibility of a recall and social desirability bias cannot be excluded. Fourth, the cross-sectional design of this study precludes causal inferences in interpreting our findings. This study did not include data of food frequency questionnaires based on results from a previous study that found no significant association between dietary Zn intake and serum Zn concentration [23]. Despite these limitations, our study has several notable strengths. The study uses a nationally representative dataset, has a large sample size, and the survey questions were extensively tested, thus increasing their validity. The availability of several covariates for adjustment, including serum albumin and hemoglobin levels, is another strength.

**In conclusion**, this secondary analysis of the NHANES 2011–14 data reveals an overall significant association between serum Zn levels and basic physical functioning difficulty in a nationally representative sample of US adults aged 50 years and older. Men in the high Zn group had lower odds of functioning difficulty, whereas women in the middle Zn group had higher odds of difficulty compared to their respective low Zn groups. The association between serum Zn groups and physical functioning difficulty also varied by educational attainment status. Further studies are needed to confirm our findings and to uncover plausible mechanisms or underlying associated factors.

## Supplementary Information

**Additional file 1: Appendix Table 1A.** The distribution of basic physical functioning difficulty in continuous variables (coded as 0 to 12) of NHANES 2011–14 participants aged 50 and older (total *N* = 1136). **Appendix Table 1B.** The distribution of counts in difficulty of basic physical functioning in four aspects (feeding, dressing, transferring, and walking) of NHANES 2011–14 participants aged 50 and older (total N = 1136). **Appendix Table 2.** Multivariate logistic regression analysis of factors associated with basic physical functioning difficulty in the 2011–14 NHANES data stratified by sex.

## Data Availability

Those who would like to access the dataset and study materials can reach Dr. Haile at haile@ohio.edu or Dr. Chavan chavan@ohio.edu, or access the National Center for Health Statistics website https://www.cdc.gov/nchs/nhanes/Index.htm.

## Abbreviations

ADL: activities of daily living
aOR: adjusted odds ratio
BMI: body mass index
CI: confidence interval
NHANES: National Health and Nutrition Examination Survey
OR: odds ratio
SE: standard error
Zn: zinc
