# Supplementary material for: Association between serum zinc levels and basic physical functioning: secondary data analysis of NHANES 2011–14
Source: BMC Nutr. 2021 Oct 11;7:57. doi: 10.1186/s40795-021-00461-z (PMC8504005; doi:10.1186/s40795-021-00461-z)
Supplement: Supplementary file 1 — Additional file 1: Appendix Table 1A. The distribution of basic physical functioning difficulty in continuous variables (coded as 0 to 12) of NHANES 2011–14 participants aged 50 and older (total N = 1136). Appendix Table 1B. The distribution of counts in difficulty of basic physical functioning in four aspects (feeding, dressing, transferring, and walking) of NHANES 2011–14 participants aged 50 and older (total N = 1136). Appendix Table 2. Multivariate logistic regression analysis of factors associated with basic physical functioning difficulty in the 2011–14 NHANES data stratified by sex. [file 40795_2021_461_MOESM1_ESM.docx]

| **Appendix table 1A: The distribution of basic physical functioning difficulty in continuous variables (coded as 0 to 12) of NHANES 2011-14 participants aged 50 and older (total N=1136)** | | |
| --- | --- | --- |
| Basic physical functioning difficulty (in four aspects) | Number (N) | Percentage (of 1136) |
| 0 | 853 | 75.09% |
| 1 | 113 | 9.95% |
| 2 | 81 | 7.13% |
| 3 | 32 | 2.82% |
| 4 | 22 | 1.94% |
| 5 | 15 | 1.32% |
| 6 | 12 | 1.06% |
| 7 | 3 | 0.26% |
| 8 | 3 | 0.26% |
| 9 | 0 | 0% |
| 10 | 1 | 0.09% |
| 11 | 0 | 0% |
| 12 | 1 | 0.09% |

| **Appendix table 1B: The distribution of counts in difficulty of basic physical functioning in four aspects (feeding, dressing, transferring, and walking) of NHANES 2011-14 participants aged 50 and older (total N=1136)** | | |
| --- | --- | --- |
| Counts of difficulty in four aspects of basic physical functioning | Number (N) | Percentage (of 1136) |
| 0 | 853 | 75.09% |
| 1 | 129 | 11.36% |
| 2 | 83 | 7.31% |
| 3 | 41 | 3.61% |
| 4 | 30 | 2.64% |

| **Appendix table 2:** Multivariate logistic regression analysis of factors associated with basic physical functioning difficulty in the 2011–14 NHANES data stratified by sex | | |
| --- | --- | --- |
| **Variables** | **Male**  (physical difficulty: yes=133, No=428); aOR (95% CI) | **Female**  (physical difficulty: yes=150, No=425); aOR (95% CI) |
| **Serum Zn tertile** |  |  |
| High vs. low  Middle vs. low | 0.43 (0.25 – 0.76)*  0.73 (0.35 – 1.52) | 1.42 (0.76 – 2.65)  2.67 (1.58 – 4.50)* |
| **Age (years)** | 0.98 (0.94-1.02) | 0.97 (0.94-0.997)* |
| **BMI category** |  |  |
| Obese vs. normal | 1.86 (1.05 - 3.32)* | 1.51 (0.73 - 3.14) |
| Overweight vs. normal | 1.63 (0.80 – 3.35) | 1.00 (0.45 – 2.24) |
| Under-weight vs.  normal | 0.90 (0.03 – 30.70) | 1.45 (0.43 – 4.91) |
| **Race/ethnicity** |  |  |
| Non-Hispanic Black vs. White | 0.44 (0.23 – 0.84)* | 1.46 (0.72 – 2.97) |
| Others vs. Non-Hispanic White | 0.55 (0.29 – 1.04) | 1.71 (1.07 – 2.73)* |
| **Education** |  |  |
| High School vs. < HS | 3.63 (1.54 – 8.54)* | 0.50 (0.23 – 1.06) |
| > HS vs. < HS | 0.93 (0.46 – 1.86) | 0.82 (0.48 – 1.42) |
| **Insurance**  No vs. Yes | 2.21 (0.98 – 4.96) | 0.32 (0.12 – 0.84)* |
| **Drinker vs. non-drinker** | 0.82 (0.48 - 1.39) | 0.70 (0.36 - 1.36) |
| **Smoking status** |  |  |
| Current vs. never | 1.80 (0.87 – 3.74) | 3.26 (1.56 – 6.79)* |
| Former vs. never | 1.45 (0.72 – 2.92) | 1.22 (0.61 – 2.45) |
| **Physical activity**  Non-sedentary vs. sedentary | 0.35 (0.17 – 0.73)* | 0.76 (0.42 - 1.35) |
| **Diabetes**  Yes vs. No | 1.14 (0.52 - 2.50) | 2.27 (1.24 – 4.15)* |
| **Serum albumin (gm/dL)** | 1.53 (0.65 – 3.60) | 0.42 (0.15 - 1.14) |
| **Hemoglobin** **(gm/dL)** | 0.65 (0.54 – 0.80)* | 0.92 (0.77 – 1.09) |

* Indicates a significant association with p values <0.05.

† Variables except age, hemoglobin, and serum albumin level were dichotomized as 1=yes, 0=no (baseline).

Abbreviations:  CI, confidence interval; aOR, adjusted odds ratio.
